# DINC: A new AutoDock-based protocol for docking large ligands

**DOI:** 10.1186/1472-6807-13-S1-S11

**Published:** 2013-11-08

**Authors:** Ankur Dhanik, John S McMurray, Lydia E Kavraki

**Affiliations:** 1 Department of Computer Science, Rice University, Houston, Texas, 77005, USA; 2 Department of Experimental Therapeutics, UT MD Anderson Cancer Center, Houston, Texas, 77054, USA; 3 Department of Bioengineering, Rice University, Houston, Texas, 77005, USA

## Abstract

**Background:**

Using the popular program AutoDock, computer-aided docking of small ligands with 6 or fewer rotatable bonds, is reasonably fast and accurate. However, docking large ligands using AutoDock's recommended standard docking protocol is less accurate and computationally slow.

**Results:**

In our earlier work, we presented a novel AutoDock-based incremental protocol (DINC) that addresses the limitations of AutoDock's standard protocol by enabling improved docking of large ligands. Instead of docking a large ligand to a target protein in one single step as done in the standard protocol, our protocol docks the large ligand in increments. In this paper, we present three detailed examples of docking using DINC and compare the docking results with those obtained using AutoDock's standard protocol. We summarize the docking results from an extended docking study that was done on 73 protein-ligand complexes comprised of large ligands. We demonstrate not only that DINC is up to 2 orders of magnitude faster than AutoDock's standard protocol, but that it also achieves the speed-up without sacrificing docking accuracy. We also show that positional restraints can be applied to the large ligand using DINC: this is useful when computing a docked conformation of the ligand. Finally, we introduce a webserver for docking large ligands using DINC.

**Conclusions:**

Docking large ligands using DINC is significantly faster than AutoDock's standard protocol without any loss of accuracy. Therefore, DINC could be used as an alternative protocol for docking large ligands. DINC has been implemented as a webserver and is available at http://dinc.kavrakilab.org. Applications such as therapeutic drug design, rational vaccine design, and others involving large ligands could benefit from DINC and its webserver implementation.

## Background

Modeling the structure of a protein-ligand complex is important for understanding the binding interactions between a potential medicinal compound (the ligand) and its therapeutic target (the protein). Moreover, such modeling aids in evaluating the thermodynamic stability of the complex. Computer-aided docking [1-4] is a technique that explores the motion space of the protein-ligand complex in order to compute energetically stable conformation(s) that model(s) the structure of the complex. In general, the exploration of the motion space is done by a sampling algorithm and the stability of a conformation of the complex is evaluated using a scoring or energy function that estimates the binding affinity of the complex. Several methods/programs have been developed for computer-aided docking (for example, [5-13]). Most docking programs treat the protein as a rigid structure and explore only the motion space of the ligand, which is composed of the rotational degrees of freedom (DoFs) of the ligand, and the translational and orientational DoFs. Docking small ligands with 6 or fewer rotatable bonds is in general very fast and accurate [14,15]. However, as the dimensionality of the motion space increases with large ligands, fast and accurate docking becomes very challenging.

Tackling the challenge of docking large ligands is important for designing putative drug compounds that have many rotatable bonds. Peptides or peptidomimetics [16,17], which are essentially small chains of natural or modified amino acids connected together with peptide bonds, are one such class of compounds. Drug design based on the peptides or peptidomimetics is rapidly gaining traction in the pharmaceutical industry [18]. These compounds are becoming popular because of their low toxicity and high specificity. Interest in these compounds has also increased with the development of sophisticated manufacturing techniques. The number of peptides authorized by the United States Food and Drug Administration is increasing at an annual rate of 8% and it is projected that the market for the peptide-based drugs will be huge [19]. Clearly, accurate and fast docking of peptides and peptidomimetic compounds will be very useful.

A method for accurate and fast docking of large ligands could also be useful for rational vaccine design. Recognition of epitopes or peptide fragments (from antigenic proteins) bound to Major Histocompatibilty Complex (MHC) molecules triggers T-cells mediated immune response. Predicting the peptide fragments that bind to the MHC molecules is crucial for developing antigen-specific vaccines [20,21]. Computational prediction of the peptide fragments that bind to the MHC molecules is thus an active area of research [22,23]. Since a large number of peptide sequences and MHC molecules can potentially interact and form complexes leading to the immune response, there exists a pressing need for a computationally fast and accurate method for docking large ligands such as the peptide fragments.

Docking of large ligands such as peptides has been a focus of some methods (e.g., [22-25]). Tong et al.'s method [22] first docks two anchor residues corresponding to each end of the peptide and then uses loop closure [26] to compute the positions of the rest of the residues. The pDock method [23] uses the ICM docking program [6] to dock the peptide and a Monte Carlo procedure to refine the docked conformation of the peptide. Computational methods such as those by Sood et al. [27] and Raveh et al. [28] are aimed at de-novo design and docking of peptides, and use peptide fragments from the Protein Data Bank (PDB) [29] to build the novel peptide. The Viterbi algorithm for de-novo peptide design [30] places residue pairs on a pre-determined path in the binding cavity of the target protein and then docks the residue pairs using AutoDock [9]. Molecular Dynamics based approaches for protein-peptide docking have also been proposed [31,32]. Although the methods described above have proven successful, they do not provide a general framework for docking large ligands as they make use of specific assumptions. For example, in the method by Tong et al. [22], it is assumed that the binding sub-pockets, where the anchor residues will bind, are approximately known. Other methods such as those based on Molecular Dynamics are computationally slow.

Our strategy for docking large ligands does not require us to make any assumptions about specific binding interactions (although such assumptions can be incorporated) and we are able to expedite computation time. We rely on the general docking framework of AutoDock [9,33] which is an excellent, widely used non-commercial docking program. AutoDock typically performs a genetic algorithm based stochastic exploration of the motion space of a ligand while simultaneously minimizing an empirical scoring function. AutoDock docks small ligands, with 6 or fewer rotatable bonds, in an accurate and fast manner [15]. However, as a ligand becomes larger, the exploration of the motion space becomes more challenging and the accuracy deteriorates [14]. To improve accuracy, AutoDock's standard protocol for docking large ligands recommends a more exhaustive exploration of the motion space. This exhaustive exploration results in improved accuracy, but also a significant increase in the computational time. In our earlier work [34], we described an incremental docking protocol, henceforth called DINC, which was designed to address the limitations of AutoDock when docking large ligands. The incremental strategy adopted by DINC is similar in spirit to that used by several previously published docking methods [8,10,35-41]. DINC performs docking using AutoDock incrementally instead of in one single step. First, a fragment of the ligand is selected. It is then repeatedly docked and extended until all of the atoms comprising the ligand are docked. At each incremental step, AutoDock is used to dock a small subset of the bonds (and associated atoms) of the ligand and, thus, instead of exploring the full motion space of the ligand in one single step, DINC explores, at each increment, only a low-dimensional subspace of the full motion space. Since AutoDock is fast and accurate when docking a small ligand with a small number of rotatable bonds, DINC results in computationally fast docking of a large ligand by dividing the docking problem into smaller sub-problems.

This paper presents a detailed analysis of the docking performance of DINC and compares it with the docking performance of AutoDock's standard protocol. Three specific docking examples involving large ligands are presented which showcase different aspects of DINC. The results from an extended docking study are also presented. We also show that, when computing a docked conformation, DINC can also be used to restrain any part of the ligand to a specific binding sub-pocket based on either biological evidence or hypotheses related to specific binding interactions. We also show that in a docking application involving a large ligand, DINC can be used to quickly compute a docked conformation of the ligand which can then be refined. We finally introduce a webserver that is designed for docking large ligands with more than 6 rotatable bonds. The webserver uses DINC for docking and also includes the extension for setting up positional restraints. The analysis of the docking performance presented in this paper shows that docking of large ligands using DINC is significantly faster than AutoDock's standard protocol. Moreover this computational speed-up is achieved without sacrificing the docking accuracy that is obtained using the standard protocol.

## Results and Discussion

In our earlier work [34], we presented an AutoDock-based [9,33] incremental protocol (DINC) for docking large ligands. The central idea of DINC is to use AutoDock, in each incremental step, for exploring a maximum of 6 rotatable bonds of a large ligand. This is done because AutoDock is fast and accurate when exploring motion spaces that are low-dimensional. DINC proceeds in multiple steps until all the rotatable bonds of a large ligand are explored. In the first step, a fragment of the ligand comprising of 6 rotatable bonds and atoms directly moved by rotations around those bonds is picked. The fragment is docked using AutoDock, and a few conformations of the docked fragment are selected and then extended by adding a small number of rotatable bonds and atoms. The extended fragments are docked, a few conformations are selected and extended, and the process is repeated until no unexplored bonds remain. Here we present a detailed analysis of the docking performance of DINC, show how DINC can be used to apply restraints on the ligand while docking, and introduce a webserver.

### Three representative examples

We first present results obtained from the docking of three large ligands to their respective target proteins. Each ligand was docked to the target protein using both DINC and AutoDock's standard docking protocol. The docking results illustrate the strengths and weaknesses of both docking protocols. Note that in this paper we focus on protein-ligand complexes for which experimentally derived structures are available in the PDB. This allows us to evaluate docking accuracy by computing the Root Mean Square Deviation (RMSD) between the conformation of the ligand computed by DINC and the conformation of the ligand from the PDB structure of the complex. Each docking protocol was given an unbound conformation of the ligand, the experimentally derived conformation of the target protein from the PDB structure of the protein-ligand complex, and the approximate location of the binding pocket. The binding pocket was defined by a three-dimensional rectangular box encompassing the binding pocket. Details are available in the Methods Section.

Each docking produced multiple docked conformations of the ligand as well as corresponding binding energy scores which were computed using AutoDock's scoring function. The conformations were ranked based on the scores, a lower scoring conformation was ranked higher. Since an experimentally derived conformation of the bound ligand (true conformation) is available, for each docked conformation of the ligand, a RMSD value was also computed. The RMSD value measures the distance between the docked conformation and the true conformation. The conformations were also ranked based on the RMSD values, a conformation with lower RMSD value was ranked higher. We will denote the highest ranked conformations based on the scores and the RMSD values as *Top-scoring *and *Top-RMSD *conformations respectively.

#### PDB ID 2FDP

**Figure 1:**
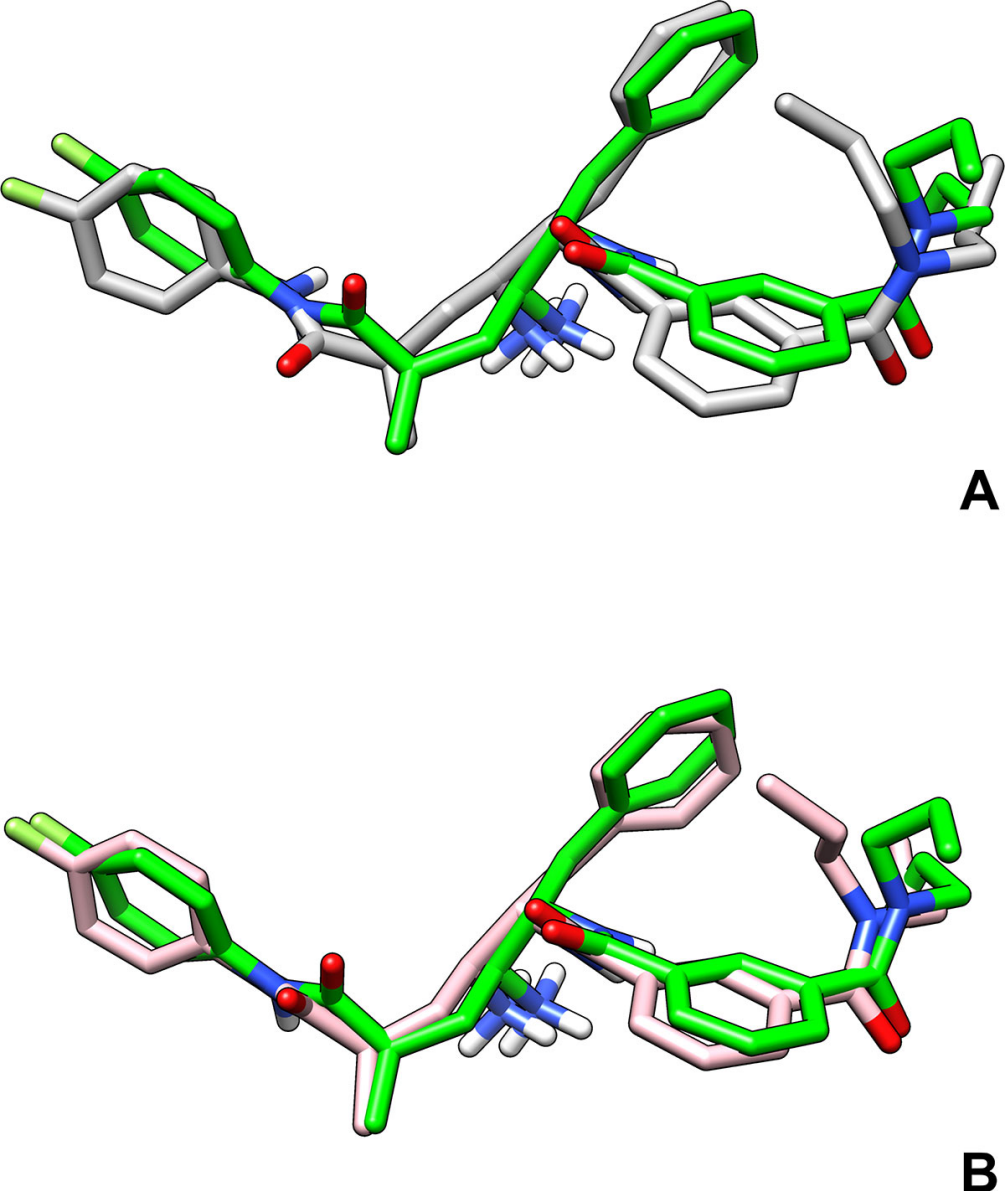
**Docking example: PDB ID **2FDP. Docking of a large ligand, with 14 rotatable bonds, to beta-secretase. The conformation of the ligand from the structure of the complex deposited in the PDB (ID 2FDP) is shown in green color. (A) *Top-scoring *conformation computed using AutoDock's standard docking protocol, (B) *Top-scoring *conformation computed using DINC.

This example illustrates the main strength of DINC: its ability to compute docked conformations in significantly shorter time with accuracy comparable to that achieved using AutoDock's standard protocol. The structure of the protein-ligand complex deposited in the PDB with ID 2FDP[42] contains a potential inhibitor of BACE-1 (beta amyloid precursor protein cleaving enzyme). BACE-1 is a beta-secretase implicated in Alzheimer's disease which is associated with deposition of amyloid-*β *peptide in the brain, and leads to the loss of brain function in Alzheimer's patients [43]. Inhibition of BACE-1 is therefore an important goal of the drug discovery community [44]. The potential inhibitor is a large ligand with 14 rotatable bonds. Docking of the ligand using AutoDock's standard docking protocol resulted in a *Top-scoring *conformation of the ligand that is at a RMSD distance of 1.43Å from the true conformation (see Figure 1A). The *Top-scoring *conformation of the ligand obtained using DINC is at a RMSD distance of 1.16Å (see Figure 1B). Thus, both protocols computed very accurate conformations with DINC performing slightly better. However, the strength of DINC is that it is significantly faster than the standard protocol: while the standard protocol used 9.77h to perform the docking, the docking time used by DINC was 0.45h. Note that all docking times are total CPU times, unless otherwise stated. Due to parallel implementation, the actual time used by DINC was 0.09h.

#### PDB ID 2ER9

**Figure 2:**
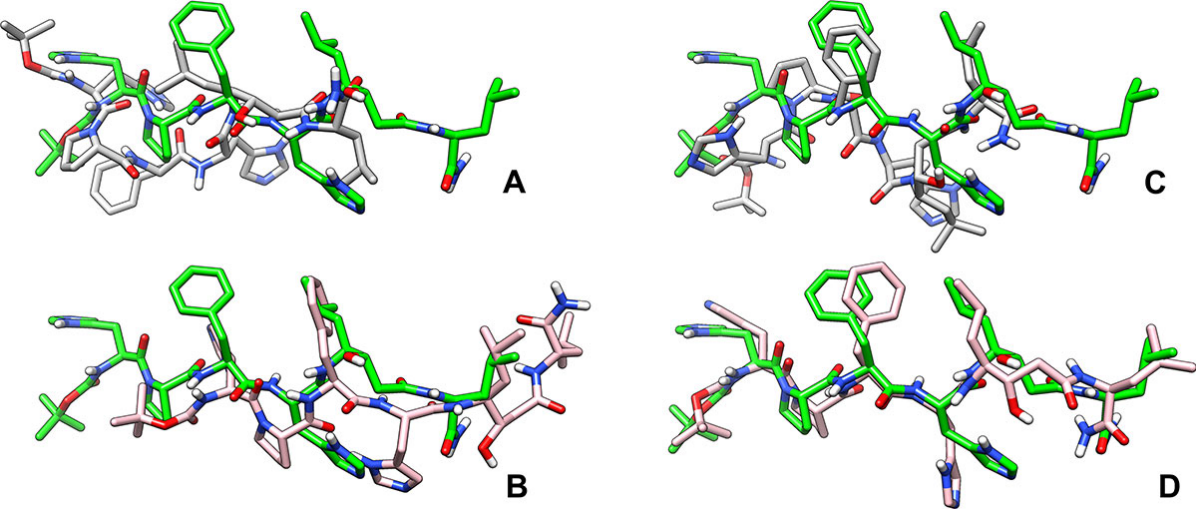
**Docking example: PDB ID **2ER9. Docking of a large ligand, with 25 rotatable bonds, to aspartic proteinase. The conformation of the ligand from the structure of the complex deposited in the PDB (ID 2ER9) is shown in green color. (A) *Top-scoring *conformation computed using AutoDock's standard docking protocol, (B) *Top-scoring *conformation conformation computed using DINC, (C) *Top-RMSD *conformation computed using AutoDock's standard docking protocol, (D) *Top-RMSD *conformation computed using DINC.

This example illustrates limitations of the scoring function and how these limitations affect docking. The scoring function is a major component of any computational prediction method as it provides a measure of the quality of prediction. Docking is no exception [45]. The sampling algorithm of a docking method explores the motion space of the ligand and computes many conformations. If a computed conformation is close to an experimentally observed one, then such a conformation can be identified only if the scoring function ranks it higher than the rest of the computed conformations. The structure deposited in the PDB with ID 2ER9[46] contains a statin-based inhibitor complexed with an aspartic proteinase. The inhibitor was designed to study the binding of such statin-based inhibitors to the aspartic proteinases, with the larger goal of achieving inhibition of the plasma proteinase renin for the purposes of lowering blood pressure in humans [47], thus, leading to the treatment of hypertension. The inhibitor is a very large ligand with 25 rotatable bonds. Docking of the ligand using AutoDock's standard docking protocol resulted in a *Top-scoring *conformation of the ligand that is at a RMSD distance of 6.57Å from the true conformation (see Figure 2A). The *Top-scoring *conformation of the ligand obtained using DINC is at a RMSD distance of 6.59Å (see Figure 2B). Figures 2A and 2B show that although the RMSD distances are similar, the *Top-scoring *conformation computed by DINC is qualitatively more accurate. The conformation computed by DINC overlaps well with the true conformation except that they are slightly offset from each other in the rigid body translation space. As expected the docking time used by DINC (1.32h) was significantly lower than that used by the standard protocol (23.35h).

It is interesting to note that comparison of the *Top-RMSD *conformations computed by DINC and the standard protocol, shows that DINC computed a conformation that was very close (RMSD = 1.87Å) to the true conformation (see Figure 2D). On the other hand, *Top-RMSD *conformation computed by the standard protocol was at a RMSD distance of 5.52Å from the true conformation (see Figure 2C). Thus, both protocols computed more accurate (RMSD-wise) *Top-RMSD *conformations as compared to the *Top-scoring *conformations. However, AutoDock's scoring function did not rank the *Top-RMSD *conformations higher than the *Top-scoring *conformations. Comparison of the RMSD values corresponding to the *Top-RMSD *conformations computed by DINC and the standard protocol shows that DINC clearly performed much better in this example.

#### PDB ID 1NDZ

**Figure 3:**
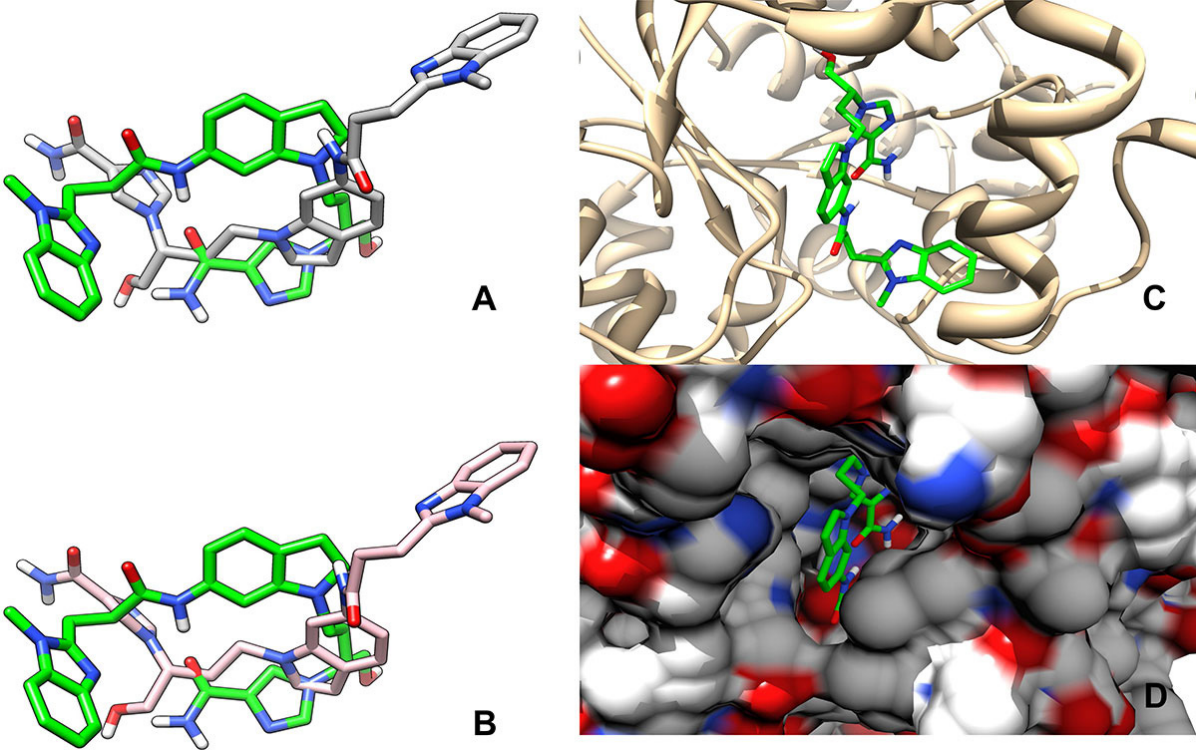
**Docking example: PDB ID **1NDZ. Docking of a large ligand, with 10 rotatable bonds, to adenosine deaminase. The conformation of the ligand from the structure of the complex deposited in the PDB (ID 1NDZ) is shown in green color. (A) *Top-scoring *conformation computed using AutoDock's standard docking protocol, (B) *Top-scoring *conformation computed using DINC, (C) Structure deposited in the PDB with the protein shown in stick representation (D) Structure deposited in the PDB with the protein shown in surface representation.

This example represents a very challenging docking problem. The structure of the complex deposited in the PDB with ID 1NDZ[48] contains a ligand with 10 rotatable bonds in complex with adenosine deaminase which is an enzyme that is found in almost all human tissues. It is involved in purine metabolism [49] and is implicated in various immune system related diseases, including psoriasis, rheumatoid arthritis, and others [50]. The large ligand in this example is a highly potent inhibitor of adenosine deaminase. Docking of the ligand using AutoDock's standard docking protocol resulted in a *Top-scoring *conformation of the ligand that is at a RMSD distance of 9.62Å from the conformation deposited in the PDB (see Figure 3A). The *Top-scoring *conformation of the ligand obtained using DINC is at a RMSD distance of 9.71Å (see Figure 3B). The docking times required by DINC and the standard protocol were 0.29h and 8.10h respectively.

Even though DINC was significantly faster, the conformations obtained using both protocols were not accurate. The inaccuracy is a direct consequence of a well-known limitation of rigid docking programs [45,51,52]. Such programs do not account for protein flexibility and treat the protein as a rigid molecule. When the binding site of the protein deforms, docking with the rigid docking programs becomes challenging. This is the case with the protein-ligand complex 1NDZ. Docking accuracy suffers because the ligand is deeply buried in the binding site (see Figures 3C, D) which undergoes a conformational change upon binding that DINC, AutoDock, as well as other docking studies [48] are not able to predict. Thus, even though docking was not successful using DINC, its computational efficiency ensures that such difficult docking scenarios could be quickly identified. On the other hand, docking using the standard protocol, in this particular example, wastes the computational resources on a problem that is not tractable using rigid body docking.

### Extended docking study

To more comprehensively evaluate the docking performance of DINC, we conducted an extended docking study. Five repeated docking experiments were performed on a dataset of 73 protein-ligand complexes compiled from the core set of the PDBbind database [53]. The 73 selected complexes have ligands with more than 6 rotatable bonds. In each docking experiment 73 ligands were docked to their respective proteins using DINC as well as AutoDock's standard protocol. The details of the docking experiment are presented in the Methods Section as well as in our earlier work [34]. Here we present the results from the docking performance evaluation and compare DINC with AutoDock's standard protocol. The following docking performance metrics were evaluated based on the docking results from each experiment:

• *DT *which represents the total CPU time, averaged over the 5 repeated docking experiments, spent in docking the 73 ligands in a docking experiment,

• *R^CS ^*which represents the RMSD value, averaged over the 5 repeated docking experiments, corresponding to the *Top-scoring *conformation of a docked ligand,

• $R_{a}^{C\;S}$ which represents the average of 73 *R^CS ^*values corresponding to the 73 ligands that were docked,

• *R^CR ^*which represents the RMSD value, averaged over the 5 repeated docking experiments, corresponding to the *Top-RMSD *conformation of a docked ligand,

• $R_{a}^{C\;R}$ which represents the average of 73 *R^CR ^*values corresponding to the 73 ligands that were docked.

A comparison of *DT *values corresponding to the docking experiments done using DINC and AutoDock's standard protocol shows that DINC is significantly fast. While the standard protocol took 725.85 hours of total CPU time to dock 73 large ligands, DINC took 31.70 hours; DINC was able to dock approximately 23 times faster than the standard protocol. As described later in the Methods Section, DINC is easily parallelized. With a parallelized implementation, DINC is up to 2 orders of magnitude faster. Thus, use of DINC results in a massive increase of computational speed. Although computational methods usually entail a trade-off between computational speed and accuracy, our results show that in the case of DINC increase in computational speed is obtained without sacrificing accuracy.

**Figure 4:**
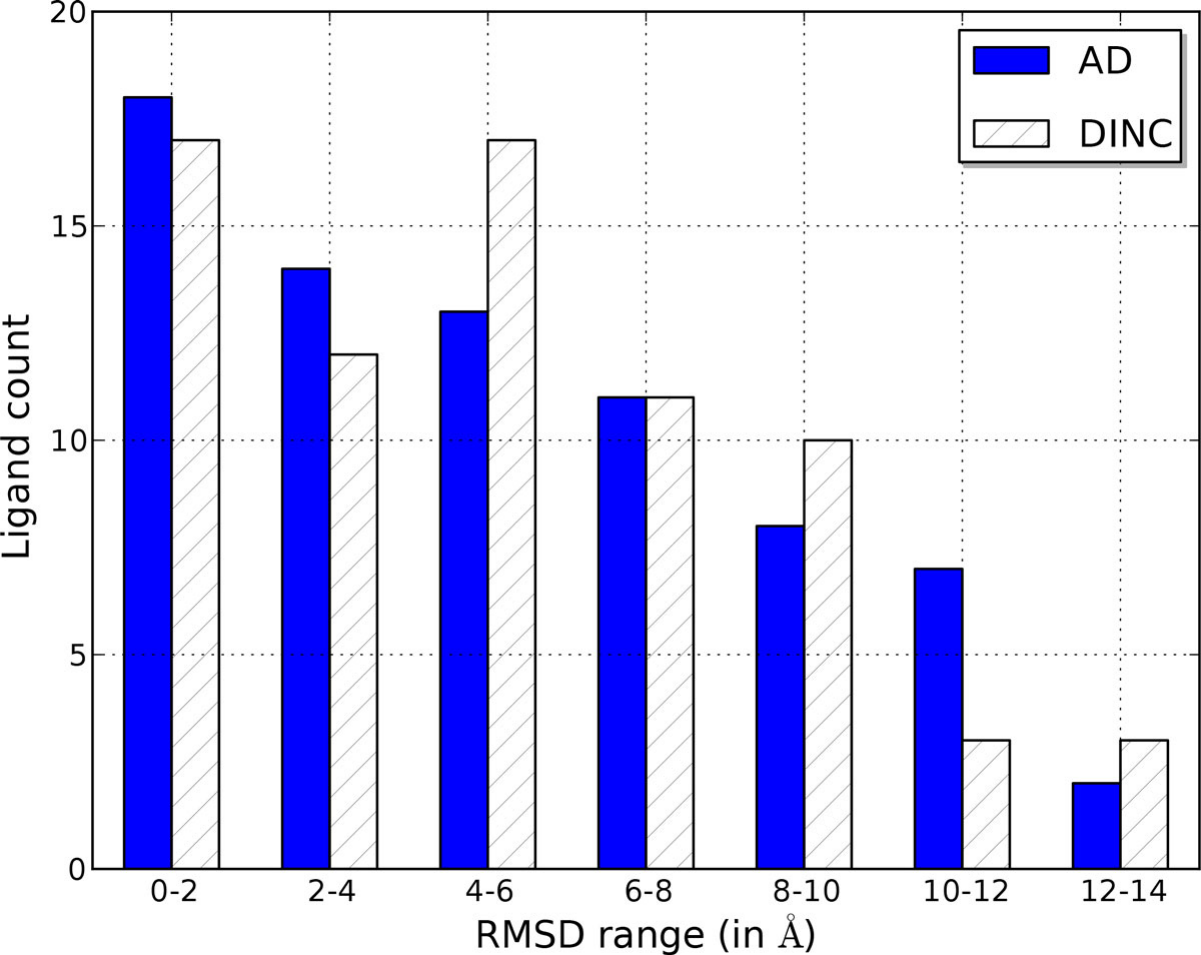
**RMSD values of Top-scoring docked conformations**. Distribution of *R^CS ^*RMSD values obtained through docking experiments done using DINC and AutoDock's standard protocol. This figure appeared in [34].

The accuracy of a docking program is measured by its ability to sample a docked conformation of the ligand that is spatially close to the true conformation of the ligand from the experimentally derived structure of the protein-ligand complex and by its ability to assign a low score to the docked conformation, ideally the lowest score among all the sampled conformations. Figure 4 compares the docking accuracy of DINC and AutoDock's standard protocol. The figure shows the distribution of 73 *R^CS ^*values corresponding to the lowest scoring docked conformations of the 73 ligands. The overall distributions are similar for both protocols which proves that the docking accuracy of the two protocols is similar. This is also reflected by the $R_{a}^{C\;S}$ values (5.06Å for DINC and 5.17Å for AutoDock's standard protocol). In the case of the docking of a small ligand, a docked conformation that is within 2Å RMSD of the true conformation is considered very accurate. In the case of docking a large ligand, the accuracy criterion is sometimes relaxed [54,55]. However, it is clear from Figure 4 that the number of *Top-scoring *docked conformations that are acceptably accurate (RMSD *≤ *4Å) is low.

**Figure 5:**
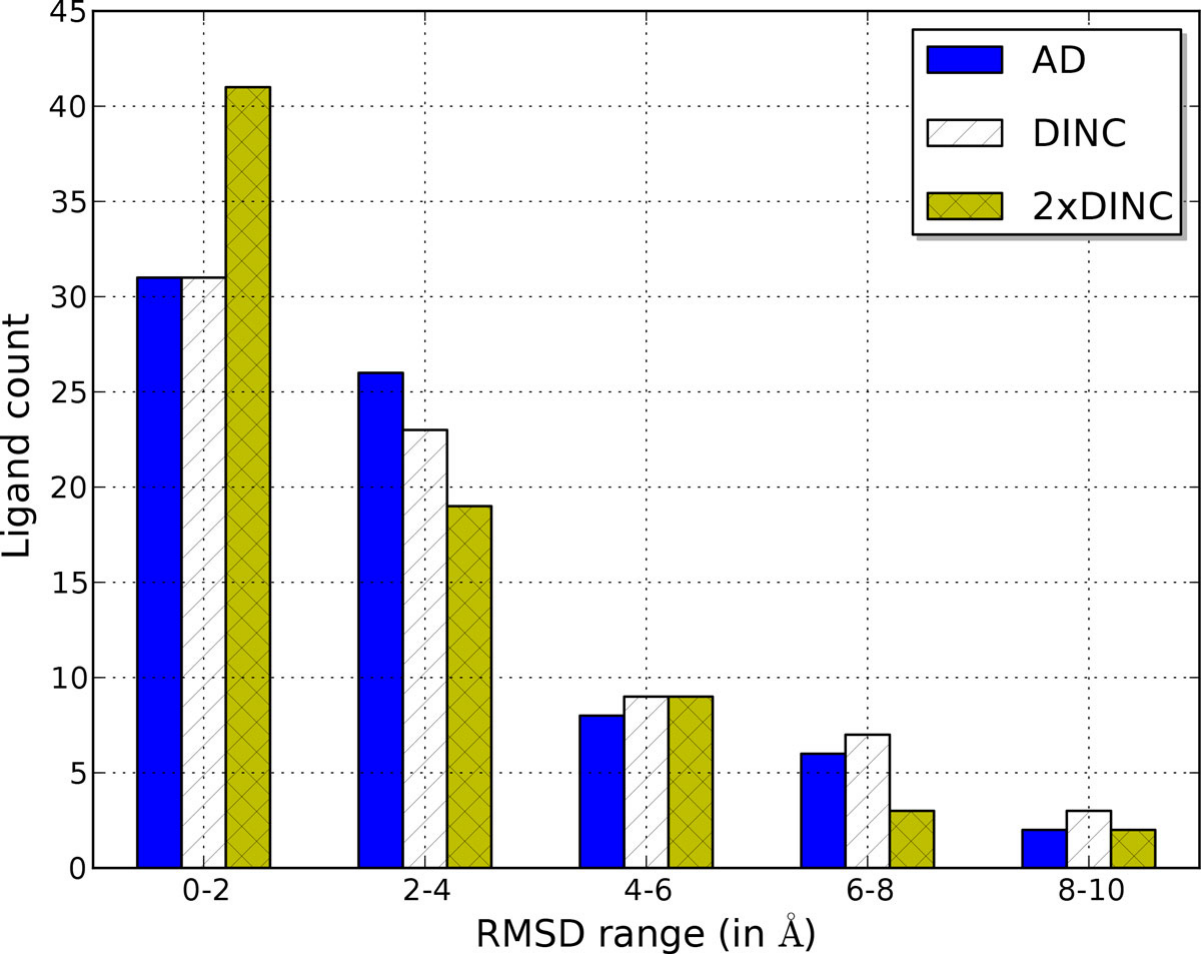
**RMSD values of Top-RMSD docked conformations**. Distribution of *R^CR ^*RMSD values obtained through docking experiments done using DINC, AutoDock's standard protocol, and through a docking experiment that combined docking results from 2 runs of DINC. This figure appeared in [34].

The low number of accurate conformations can be due to two reasons: (a) an accurate docked conformation is not sampled due to insufficient exploration of the motion space, (b) an accurate docked conformation, although sampled, is not assigned the lowest score due to insufficient scoring function. To further investigate the reasons for the few acceptably accurate conformations, we analyzed the *R^CR ^*values. These values correspond to the most accurate docked conformation of the ligand which might or might not be the lowest scoring conformation. A distribution plot of the *R^CR ^*values computed from the docking experiments using DINC and AutoDock's standard protocol is shown in Figure 5. The distribution of the *R^CR ^*values is similar for both protocols as also reflected by the $R_{a}^{C\;R}$ values (3.01Å for DINC and 2.92Å for AutoDock's standard protocol). However, a comparison of distributions shown in Figures 4 and 5 illustrates that the number of docked conformations with low *R^CR ^*values is higher than the number of docked conformations with low *R^CS ^*values. In half of the cases for which an acceptably accurate docked conformation was sampled, it was not identified by AutoDock's scoring function as the lowest scoring conformation. The limitation of AutoDock's scoring function when estimating the binding affinity of complexes involving large ligands is, thus, evident. But the limitation of the scoring function is not the only reason for the low number of accurate conformations. Insufficient exploration of the motion space of the ligand is the other reason. Figure 5 also shows the distribution of the best results obtained after combining the results from two docking experiments that were done using DINC. Note that repeated experiments using DINC produced different results because of the stochasticity inherent in DINC. The distribution clearly illustrates the noticeable increase in the number of ligands for which acceptably accurate docked conformations were sampled. The two docking experiments combined still took an order of magnitude lesser computational time than the docking experiment done using AutoDock's standard protocol. Thus, DINC's advantage is that it can more exhaustively explore the motion space of the ligand, and it does so in significantly less time than AutoDock's standard protocol.

### Restraints and molecular dynamics

A useful feature of DINC is that a positional restraint can be enforced on a part of the ligand. For example, we recently applied DINC to a modeling problem [56] involving large peptidomimetic compounds targeting the SH2 (src homology 2) domain of STAT3 (signal transducer and activator of transcription 3) [57], a protein that is implicated in a variety of human cancers [58,59]. The peptidomimetic compounds contain a PTyr-Xaa-Yaa-Gln motif and the approximate location of the phosphorus atom (or phosphate group) contained in the phosphotyrosine (pTyr) residue is known. In such cases, where there is experimental evidence or a hypothesis regarding the approximate location of an atom of the ligand, DINC can exploit the positional restraint on the atom, thereby leading to a more accurate docking performance. As described in the Methods Section, DINC docks a large ligand incrementally where at each increment, it docks a fragment and then selects a few docked conformations for further docking. Thus, we can enforce a positional restraint on an atom of the ligand, by first picking it as a root atom (see Methods Section) and then, at each increment, selecting docked conformations of the fragments based on the following modified scoring function,

(1) $$S=\;0.\text{25}\left(D_{a}\right)\;+S_{AD},$$

where, *D_a _*is the square of the Euclidean distance of the atom from its desired location, and *S_AD _*is the score computed by AutoDock's scoring function. The weight for *D_a _*has been assigned such that a large distance (*D_a _>*10Å^2^) between the atom and its desired location is penalized by 2.5kcal/mol (the standard error in AutoDock's scoring function).

To refine the structure of a protein-ligand complex obtained through docking, Molecular Dynamics simulation [60] of the protein-ligand complex is often performed. DINC provides a computationally fast way of obtaining the starting conformation for refinement using Molecular Dynamics. In the context of the modeling problem involving the peptidomimetic compounds and STAT3, we recently described a modeling strategy [56] which uses DINC for computing docked conformations of the complexes, selects the best docked conformation using the scoring function described by equation 1, and then performs the molecular dynamics simulations. Through rigorous experiments we showed [56] that the modeling strategy was able to model accurate binding modes, thus demonstrating a very useful application of DINC.

### Webserver

**Figure 6:**
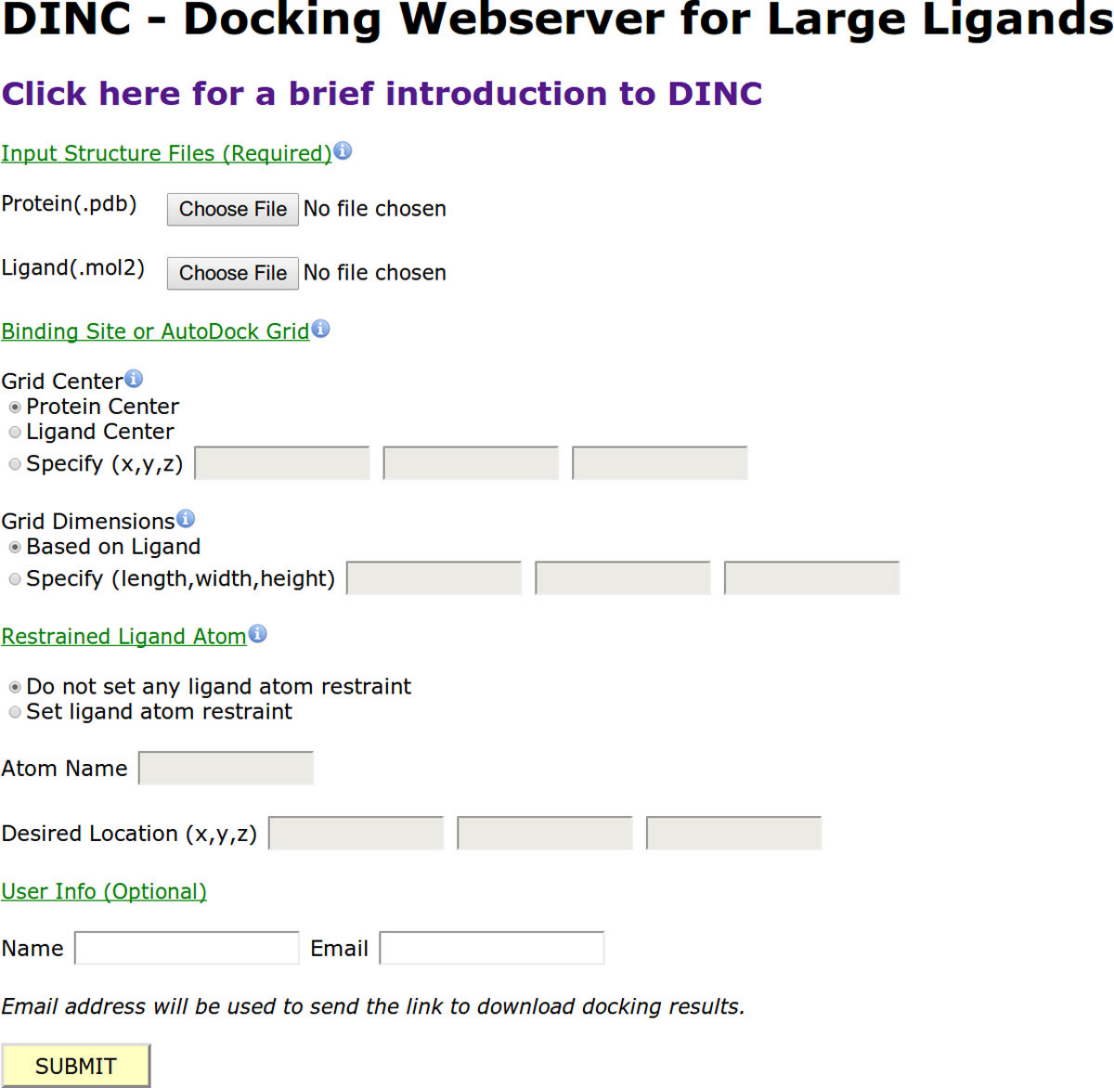
**DINC webserver**. A webserver implementation of DINC is available at http://dinc.kavrakilab.org. The webserver takes as input a protein structure in pdb format and a ligand structure in mol2 format. Center and dimensions of the AutoDock grid, that encompasses the binding site, are specified. Both can either be specified in absolute terms or by using other options. For example, the grid dimensions can be specified based on the ligand as described in the Webserver Section. An atom of the ligand can also be restrained to a desired location as explained in the Restraints and Molecular Dynamics Section. The webserver outputs six docked conformations and corresponding AutoDock scores. Three of the six conformations are the conformations with the lowest AutoDock scores. The other three conformations are the representatives of the three largest clusters of the docked conformations.

A webserver implementation of the DINC protocol is freely available at http://dinc.kavrakilab.org (see Figure 6). Although the webserver can be used for docking ligands both small and large, it is mainly aimed at users who are interested in docking large ligands with more than 6 rotatable bonds. The webserver can be used to quickly compute reasonably accurate docked conformations of such large ligands. The docked conformations can be used for further refinement with Molecular Dynamics [60] or can be used in a consensus docking scheme [12,61] which combines the docking results from several methods to compute a consensus docking result. The webserver can be optionally used such that DINC can restrain an atom of the ligand to a desired location during the incremental docking process.

The input to the webserver consists of a ligand structure in mol2 format and a protein structure in pdb format. To specify the binding site of the protein, the center and the dimensions of the AutoDock grid are also given as the input. The center can be the geometric center of either the ligand or the protein. It can also be specified in absolute terms. The dimensions of the grid are either specified in absolute terms or can be determined based on the ligand as described in the Methods Section. Restraints on an atom can be set up by specifying the name of the atom (as contained in the input ligand structure file) and the coordinates of the desired location of the atom. The webserver outputs six docked conformations and the corresponding AutoDock scores. Three conformations out of the six are the ones with the lowest AutoDock scores. The other three conformations represent the three largest clusters of all the docked conformations; each of the three conformations is the lowest scoring conformation in its respective cluster.

## Conclusions

Computer-aided docking of large ligands, ligands with more than 5 or 6 rotatable bonds, is challenging [15]. Docking of any ligand requires the exploration of the motion space of the ligand. When the ligand has less than 5 or 6 rotatable bonds, most of the existing programs are able to dock the ligand in a fast and accurate manner. However, for a large ligand, the increased dimensionality of the motion space makes exploration for the docked conformation challenging and computationally slow. Like any other computational method, the docking program suffers from the trade-off between computational speed and accuracy. In this paper, we showed that improvements in the computer-aided docking of large ligands can be achieved by using our AutoDock-based incremental docking protocol, DINC, and we introduced a webserver implementation of DINC (Figure 6). The DINC webserver is available at http://dinc.kavrakilab.org.

We presented a detailed analysis of the strengths and weaknesses of DINC as compared to the standard protocol recommended for docking large ligands using AutoDock. We compared the docking performance of DINC and AutoDock's standard protocol using three representative docking examples which involved large ligands with 10, 14, and 25 rotatable bonds. We also presented the results from an extended docking study. Analysis of the docking results from the three specific examples, as well as the extended study, shows that DINC is on par with AutoDock regarding the extent of the ligand's motion space exploration. Both protocols are able to sample acceptably accurate conformations. However, DINC achieves the exploration of the motion space in 2 orders of magnitude less computational time compared to the standard protocol. Another important conclusion drawn from the docking results is that, even when acceptably accurate conformations are sampled, AutoDock's scoring function does not always rank them favorably.

DINC's accuracy saw improvement when positional restraints were imposed on an atom whose approximate location is known [56]. The improvement occurs because imposition of the positional restraints reduces the volume of the motion space that is explored by DINC. DINC easily incorporates the restraint information by selecting the initial fragment such that it contains the atom to be restrained, and by modifying the conformation selection criterion. At each increment, the conformations that have low AutoDock scores and have the atom close to its desired location, are preferentially selected. The option of restraining the ligand is available through the DINC webserver.

There are several applications for which DINC and the webserver can be used. Therapeutic drug design based on large compounds [62,63] such as peptides, peptidomimetics, and others could benefit from the use of DINC. DINC can be used to quickly model the protein-ligand complex and to provide an understanding of potential binding interactions that can be exploited for improving the design of drug compounds. One such application of DINC was demonstrated in our recent work [56] on predicting binding modes of peptidomimetics in complex with a cancer target. Vaccine design is another application for which DINC could be used. Predicting the fragments of an antigenic peptide which can bind to the MHC molecules is of importance to the vaccine design process [20,21]. DINC can be used for docking the fragments of the antigenic peptide in complex with the MHC molecule. The computed structures of the peptide-MHC complexes can then be evaluated using a scoring function that is specifically designed for estimating the binding affinities of such complexes. The lowest scoring candidate fragments can then be potentially used for further development in the vaccine design process. DINC can also be used within the framework of a consensus docking [12,61] scheme which has been shown to improve docking performance. The consensus scheme combines docked conformations computed by multiple docking methods and evaluates them based on a scoring criterion that reflects the consensus between the scores generated by these methods. Quickly computed docking results using DINC could, therefore, be used in such a consensus scoring scheme along with the results from existing docking programs.

To further improve DINC, two major improvements are needed. As shown in Figures 4 and 5, for a large majority of protein-ligand complexes, acceptably accurate (RMSD *≤ *4.0Å) docked conformations were computed, but the scoring function did not rank them as the *Top-scoring *conformations. Since most of the docking programs are geared for small-molecule drug discovery, a scoring function, that is designed specifically for predicting the binding affinities of the complexes involving large ligands, is needed. Such a scoring function can be developed using empirical energy terms [13] and using statistical-regression based function approximation methods [64-66]. A major improvement is needed to model the flexibility of the target binding site; this is of critical importance when the holo and apo conformations of the binding site are significantly different [67]. In such a scenario, a rigid docking program is bound to fail as illustrated earlier in one of the representative examples (Figure 3). Accounting for protein flexibility is, thus, a major focus of our research efforts. Although there are some limitations to DINC, we have shown that it can be used in various applications to quickly explore the motion space of a large ligand and compute docked conformations efficiently.

## Methods

The AutoDock-based incremental docking protocol DINC was introduced in our earlier work [34]. Here, we first present a brief overview of DINC for completeness purpose and then, using one of the three representative docking examples discussed earlier in the Results and Discussion Section, we show how the DINC webserver docks a large ligand.

Given a ligand, a protein, and the specifications of a bounding box that encompasses the binding site, DINC first processes the ligand and the protein which primarily includes assigning bonds of the ligand as rotatable or non-rotatable, and assigning atom types and charges. DINC then computes a torsion tree in which each edge represents a rotatable bond; if an edge connects node A to node B, then node B contains the set of atoms associated with the bond (i.e., the atoms that are directly moved by the rotation around the bond). The edges of the tree are ranked by the visit order in a breadth-first traversal of the tree. The root node of the tree contains a selected root atom and the atoms connected to the root atom by a sequence of non-rotatable bonds. A fragment of the ligand is selected, which comprises the atoms in the root node as well as the atoms associated with a small number of top-ranked rotatable bonds. The fragment is docked using AutoDock with parameter *ga_num_evals *set to 250000. A few lowest scoring docked conformations of the fragment are selected and are extended by adding the next few top-ranked rotatable bonds and the associated atoms. The extended conformations are docked again. In these dockings, only the rotational DoFs corresponding to the newly added bonds and some of the bonds that existed prior to the fragment extension are explored. A few of the lowest scoring docked conformations are selected, extended, and docked again. This is repeated until all of the rotatable bonds are explored and the associated atoms are docked.

**Figure 7:**
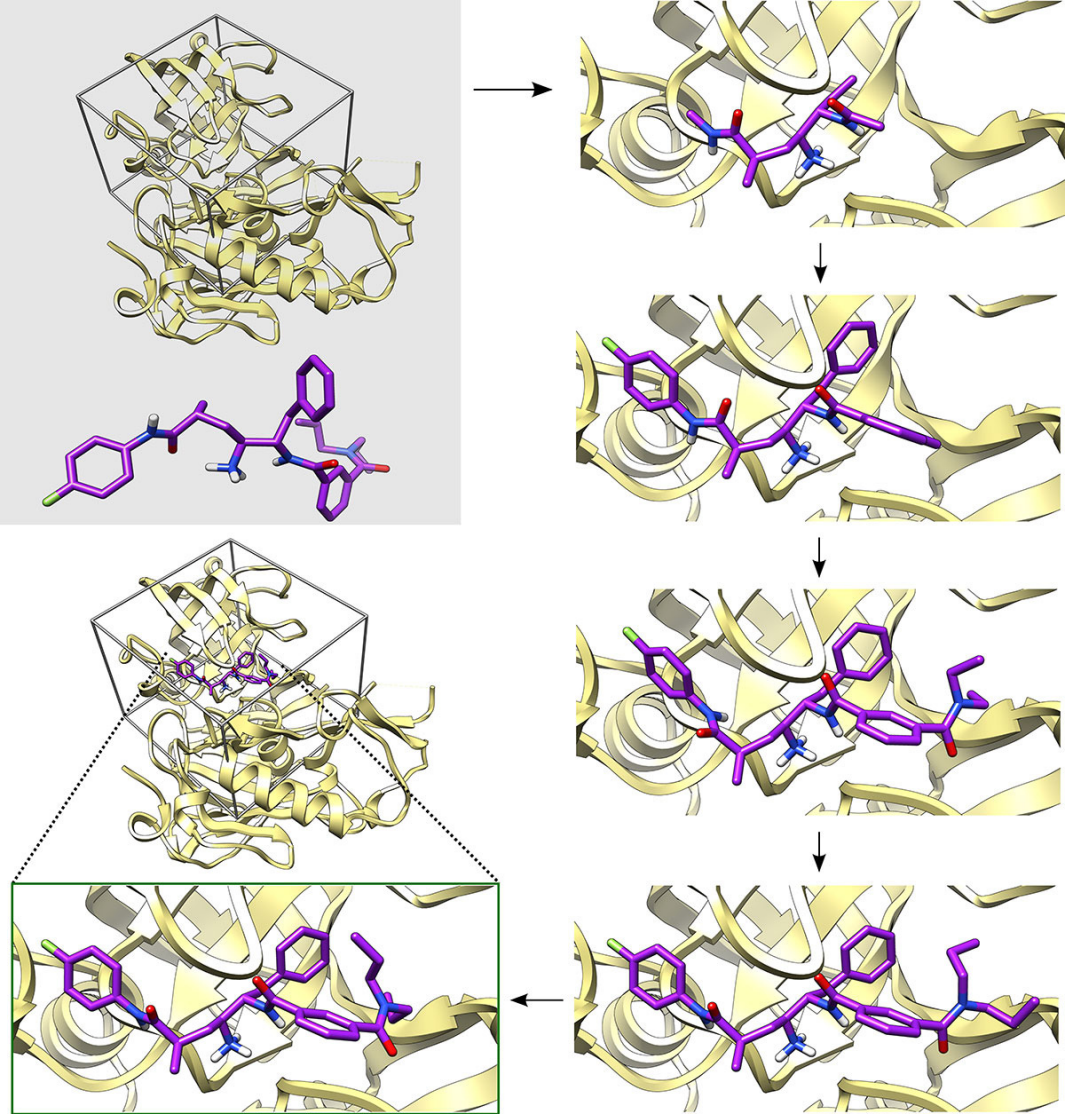
**Docking a large ligand using DINC**. Given a protein (in yellow color), a ligand (in purple color), and the approximate location of binding site (encompassed by the box), DINC docks the ligand incrementally. The protein and the ligand shown in this figure are derived from the structure of the protein-ligand complex deposited in the PDB with ID 2FDP. An initial fragment of the ligand is selected such that it has 6 rotatable bonds. The fragment is docked and 5 docked conformations are selected. These conformations are extended by adding 3 more bonds and atoms that are directly rotated by the 3 bonds. The extended conformations are docked, 5 of the docked conformations are selected and are then extended. This is repeated until the ligand is fully docked. As also shown in Figure 1, the docked conformation of the ligand is spatially close to the true conformation. For clarity, only one of the 5 selected conformations is shown at each step.

Figure 7 explains how the docking of a large ligand proceeds after a conformation of the ligand and the protein are submitted to the DINC webserver. The conformations of the ligand and the protein are derived from the structure of the protein-ligand complex deposited in the PDB with ID 2FDP. The binding site is approximated by a three dimensional rectangular box (also known as AutoDock grid) and is determined based on the true conformation of the ligand from the complex. The grid is created such that it encompasses the true conformation and is then extended along each dimension [13]. The grid is centered at the geometric center of the ligand (9.67, -2.94, 48.36) and the x, y, and z dimensions of the grid are 76, 80, and 60 respectively. Note that when the true conformation of the ligand is unknown, the binding site can be either specified in absolute terms or based on the input conformation of the protein (as described in the Webserver Section).

After processing of the ligand and the protein, the root atom is selected such that the first fragment of the ligand contains the highest number of hydrogen bond donors and acceptors combined. A random conformation of the ligand is generated so that the docking result is not influenced by the true conformation of the ligand that was input. From the ligand, a torsion tree is generated and as there are 14 rotatable bonds, they are assigned ranks 1 to 14. Atoms in the root node, plus the atoms associated with the bonds ranked from 1 to 6 are selected as the first fragment of the ligand. The first fragment is docked using AutoDock which produces 50 conformations of the first fragment in complex with the protein. Out of these 50 conformations, 5 lowest scoring conformations are selected. Each of the selected conformations is then extended by adding the atoms that are associated with the bonds ranked from 7 to 9. Now we freeze the rotational DoFs corresponding to the bonds ranked from 1 to 3, and dock the 5 extended conformations while exploring the rotational DoFs corresponding to the bonds ranked from 4 to 9. The docking of the 5 extended conformations is done in parallel for computational speed-up. Thus, we explore three newly added DoFs and re-explore three of the previously explored DoFs. The docking of each extended conformation produces 20 conformations, and out of the 100 total conformations produced, 5 lowest scoring conformations are selected. The selected conformations of the fragment are extended, and docked (in parallel) repeatedly. Thus, in two more iterations, rotational DoFs are explored for the bonds ranked from 7 to 12, and then for the bonds ranked from 10 to 14. After the DoFs corresponding to the 14 rotatable bonds are explored, we obtain 100 docked conformations of the full ligand as well as the corresponding AutoDock scores.

Each ligand in this work was docked using DINC as well as AutoDock's standard protocol. In the standard protocol, the AutoDock parameters *ga_num_evals *and *ga_run *were set to 25000000 and 50 respectively as is recommended for docking large ligands. The ligand and the protein were processed identically in dockings done using both the protocols. The 4.2 version of AutoDock was used and the experiments were done on a computing cluster (2304 total processor cores, each core runs at 2.83 GHz) at Rice University.

## Competing interests

The authors declare that they have no competing interests.

## Authors' contributions

AD, JSM, and LK developed the concepts and the method. AD developed the experiments and analyzed the data. AD, JSM, and LK interpreted the results and wrote the manuscript.

## Declarations

The publication costs for this article were funded by the corresponding author.

This article has been published as part of *BMC Structural Biology *Volume 13 Supplement 1, 2013: Selected articles from the Computational Structural Bioinformatics Workshop 2012. The full contents of the supplement are available online at http://www.biomedcentral.com/bmcstructbiol/supplements/13/S1.
